# Testing the contextual Interaction theory in a UHC pilot district in South Africa

**DOI:** 10.1186/s12913-022-07705-z

**Published:** 2022-03-15

**Authors:** Janet Michel, Nthabiseng Mohlakoana, Till Bärnighausen, Fabrizio Tediosi, David Evans, Di McIntyre, Hans T. A. Bressers, Marcel Tanner

**Affiliations:** 1 Swiss Tropical and Public Health Institute (Swiss TPH), Basel, Switzerland; 2 University of Basel, Basel, Switzerland; 3 Department of Governance and Technology for Sustainability, University of Twente, Enschede, Netherlands; 4 Professor Global Health Harvard T. H. Chan School of Public Health, Boston, United States; 5 Director Institute of Public Health, University of Heidelberg, Heidelberg, Germany; 6 Head of Health Systems and Policy Unit Swiss Tropical and Public Health Institute (Swiss TPH), University of Basel, Basel, Switzerland; 7 Swiss TPH University of Basel World Bank Health Economist, Basel, Switzerland; 8 Health Economics Unit, University of Cape Town, Cape Town, South Africa; 9 Policy Studies and Environmental Policy, University of Twente, Enschede, Netherlands

**Keywords:** Contextual interaction theory, Leadership, Motivation, Information, Resources, Context, Interactions, Implementation, Policy-practice gaps

## Abstract

**Background:**

World-wide, there is growing universal health coverage (UHC) enthusiasm. The South African government began piloting policies aimed at achieving UHC in 2012. These UHC policies have been and are being rolled out in the ten selected pilot districts. Our study explored policy implementation experiences of 71 actors involved in UHC policy implementation, in one South African pilot district using the Contextual Interaction Theory (CIT) lens.

**Method:**

Our study applied a two-actor deductive theory of implementation, Contextual Interaction Theory (CIT) to analyse 71 key informant interviews from one National Health Insurance (NHI) pilot district in South Africa. The theory uses motivation, information, power, resources and the interaction of these to explain implementation experiences and outcomes. The research question centred on the utility of CIT tenets in explaining the observed implementation experiences of actors and outcomes particularly policy- practice gaps.

**Results:**

All CIT central tenets (information, motivation, power, resources and interactions) were alluded to by actors in their policy implementation experiences, a lack or presence of these tenets were explained as either a facilitator or barrier to policy implementation. This theory was found as very useful in explaining policy implementation experiences of both policy makers and facilitators.

**Conclusion:**

A central tenet that was present in this context but not fully captured by CIT was leadership. Leadership interactions were revealed as critical for policy implementation, hence we propose the inclusion of leadership interactions to the current CIT central tenets, to become motivation, information, power, resources, leadership and interactions of all these.

## Background

Empirical research discloses implementation as a significant impediment to change [1, 2]. This paper contributes to implementation studies by testing an implementation theory, Contextual Interaction theory in a new domain, health. Our study explored policy implementation experiences of 71 actors involved in universal health coverage (UHC) policy implementation, in one South African pilot district using the CIT lens.

South Africa is the focus of this study because the South African government began piloting policies aimed at achieving UHC in 2012. Policies aimed at achieving UHC have been and are being rolled out in the selected 10 pilot districts. World-wide, there is growing UHC enthusiasm at global and national levels [3]. Everyone should be given the opportunity to live healthy productive lives, regardless of who they are or where they live. This outcome will depend to a great extent on how UHC policies are and will be implemented. For example, the South African Ministry of health`s role in providing guidance has been characterized by good policies without equivalent emphasis on implementation, monitoring and assessment of these policies through-out the system [4]. It is important therefore to bear in mind that intention of policy may not correspond with policy outputs and outcomes [2]. In 2017, the South African NHI pilot phase, health systems strengthening came to an end, though health system strengthening initiatives and improvements of service delivery platforms continue to be implemented [5]. Many lessons have been learnt during the past five years including implementation bottlenecks such as supply chain, infrastructural and resource challenges and underspending of conditional grants [3, 6]. In addition, there is a call that it is essential that once data and information from pilot districts becomes available, it should be fully utilized, evaluated and results be published to allow for government and public to engage with the process, learn as well as foster accountability [7].

### Defining implementation

To understand implementation, it is important to define the concept as used in the scope of this study. Pressman and Wildavsky [1] defined implementation as to carry out, accomplish, fulfil, produce, complete a policy. Mazmanian and Sabatier [8] define implementation as the carrying out of a basic policy decision. O`Toole [9] defines implementation as what develops between establishment of an apparent intention on the part of government to do something, or to stop doing something, and the ultimate impact in the world of action. For the purpose of this study, we define policy as translating public policy intention into results [2].

Policy implementation is a complex process incumbent upon a number of factors. It is important to not only understand why policies succeed but also why they fail [10]. Many scholars find it useful to treat implementation as a distinctive point for analysis within the policy process with an ability to shed light on the whole [1, 11, 12].Three generations of implementation theories shape the policy implementation field. The first generation, illustrated by the work of Pressman and Wildavsky is also known as top-down, second generation theories (Sabatier) [13] that focusses on bottom uppers and the third generation-a synthesis of the first two approaches. The third-generation implementation researchers see implementation as an ongoing process, regardless of result [14] and acknowledges the multiplicity of factors incumbent upon implementation including context [9, 11]. Implementation theory and research have outgrown the search for a single theory of implementation and have entered a new era that recognizes multiple theories appropriate to various implementation research questions [15].

### Justification of contextual interaction theory

Contextual Interaction Theory (CIT) is a third generation theory [9] developed in the Netherlands during the late 1990s and has been applied in several studies [2, 16] but not widely used in the health policy domain. CIT is relatively simple, with broad applicability as it analyses the very core of implementation: the motivation, information, resources and power of the policy target and implementer. CIT emphasizes the policy target and implementer, whether they exist as local implementers or higher-level administrators. A useful theory must condense reality into less detailed but informative elements [2] and CIT does exactly that, hence our choice.

Scharpf (23) writes that overly parsimonious theories ignore either actors or institutions in pursuit of the other. We selected CIT because it is parsimonious, it distils a sea of options for implementation variables into core variables of motivation, information, resources, power and interaction of these. These variable are not arbitrarily chosen as the important variables among others, but because they have high explanatory power and all form the core of policy interaction processes [2, 11]. CIT is therefore a deductive and realistic approach that allows implementation to be effectively analysed [2]. In implementation science, theoretical approaches serve three main purposes: to describe and or guide the translation of research into practice-process models, to understand or explain factors that influence policy implementation (implementation theories) and to evaluate implementation (evaluation frame works) [18]. CIT utility has been proven in all three areas elsewhere [2, 11, 19, 20] making it our theory of choice.

### Description of CIT

The theory focuses on motivation, information, power, resources and interactions of these. CIT allowed our study to focus not only on CIT variables but also context, structural and outer. This theory has been used in the South African energy context before [14]. Applying CIT to analyse UHC policy implementation is a new domain. Based on the amount of information the actors have on policy, their level of motivation, the amount of resources they have for implementation, the amount of power they have to mobilize needed resources and various interactions of these, a policy can be successfully or unsuccessfully implemented.

## Methods

### Study aim

The study aimed at tracking NHI policy implementation process through the engagement of policy makers and policy implementers in order to explore, identify and describe why and how policy-practice discrepancies come about in UHC context. Contextual interaction theory was then chosen as an analytic framework for data analysis and we took that as an opportunity to test its utility in a South African UHC context.

### Research setting and sampling

Ten pilot districts were identified by the Department of Health and selected as National Health Insurance (NHI) pilot sites. The National Department of Health (DoH) selected these sites based on poor performance on key health indicators like high maternal and child mortality rates [3]. UNITAS purposively selected three out the ten selected NHI pilot districts in South Africa. A case study design was used for this research. A case study design is defined as an empirical inquiry that investigates a phenomenon within its real-life context [21]. This study is situated in only one of the three districts, district X (name withheld for anonymity reasons). The case was the district (X), conveniently selected as the only NHI pilot district in that province at the time. Managerial willingness and support to participate in the study also guided site selection.

### The intervention

NHI piloting in South Africa started in 2012. Primary health care re-engineering and national health insurance are the two broad reforms selected to reach UHC. These comprise a suite of policies and reforms that were rolled out in selected districts. The first five years focussed on health systems strengthening, particularly, Primary Health Care. The reforms included among others, appointment of district clinical specialist teams, family-based teams, school health teams, management strengthening, referral system strengthening and the establishment of ideal clinics. The overall goal of NHI is to ensure that every South African has access to health care services of high quality, without suffering any financial impoverishment [22, 23].

### Study design and data collection

A qualitative, exploratory case study design was utilized. We tracked policy implementation aimed at achieving Universal Health Coverage in one pilot district in South Africa from 2011–2015. Data was collected during three phases 2011–2012 (Contextual mapping), 2013–2014 (Phase 1) and 2015 (Phase 2). A theory of change (TOC) approach was followed to explore universal health coverage policy implementation experiences. TOC is a theory of how and why initiatives work [24]. Theory of change describes assumptions actors have, explains steps and activities they take to achieve goals and connections between these activities and the policy outcome [24]. Semi-structured in-depth interviews were held with participants using a standard interview guide. See appendix. Participants ranged from provincial, district, sub-district and facility actors involved in policy implementation. No patients were involved since their role in policy implementation is limited. The duration of each interview varied from 2–3 h. Two researchers at every occasion, conducted the interviews in English. All participants were qualified professionals who had no problems understanding or responding in English. Full Ethical approval for the study was granted by the University of KwaZulu-Natal Biomedical Research Ethics Committee; REF BE197/13. Support letters were also provided by the provincial and district offices in our study site. All actors gave written consent and were free to withdraw from study any time [10, 25, 26].

### Data analysis

All interviews were audio-recorded. All participants gave informed and signed consent and were free to withdraw from the study at any time. An iterative, inductive and deductive data analysis approach guided by Contextual Interaction theory was utilized. Transcripts were coded with the aid of MAXQDA2018. Trustworthiness criteria were used to evaluate rigour for this study [27]. Trustworthiness concepts included dependability, credibility, confirmability and transferability. To ensure dependability we described data collection process in detail and two researchers experienced in qualitative methods, kept reflexive individual journals through-out data collection and analysis. Debriefing after interviews was done daily in the field. The two researchers further analysed the data independently before reaching consensus under the supervision of an experienced qualitative researcher. To ensure confirmability, findings were discussed with supervisors and co-authors experienced in the field, and their responses were incorporated. To enhance transferability, participants, context and process of analysis have been described in detail [27]. We achieved data saturation [28] and data source triangulation, through interviewing actors from different levels of the health system. Actor description: In a UHC pilot site the following actors are present [20].

### Justification of two actor scenario

We focussed on two actors, policy makers and policy implementers. It is worth mentioning that a multi -actor scenario in health policy evaluation is possible and appropriately suitable in cases of assessing successful policy implementation, involving policy maker, policy implementer, partners and target actors in this case patients. The focus of our study was to understand how and why policy-practice gaps come about, hence our focus is on two actors, policy maker (provincial actors) and policy implementer (district, sub-district and PHC actors) instead. We therefore tested the viability of a two-actor model-policy makers at provincial level and policy implementers (district, subdistrict, facility actors). See Table 1 below;

**Table 1:** Study actor description in general

*Policy Maker Actors*	*Linking actor*	*Implementing actors*	*Target actors*
*National DOH* *Provincial DOH*	NGOs providing training	District, subdistrict and PHC staff	Patients and communities

### Study actors

The focus of our study was to understand policy practice gaps, hence our two actors are policy maker and policy implementer, leaving out linking and target actors as they did not play an active role in UHC policy implementation. See Table 2 below:

**Table 2:** Study actor description used in our study

Policy Maker Actors	Implementing actors
National DOH Provincial DOH	District Managers and District staff Subdistrict managers and staff PHC facility staff

### Research participants

Seventy-one key informants were involved. See description in Table 3 below;

**Table 3:** Overview of key informants, research phase, role and where they worked (health system level)

Health System Level	Role	Contextual mapping	Round 1	Round 2	Total
Provincial	Policy maker -making sure NHI policies are carried out	1	1	1	**3**
District	Policy implementers ranging from district manager, programme managers, district clinical specialist team, Emergency rescue service manager and PHC supervisors with policy implementation responsibilities including the PHC supervision manual	1	5	4	**10**
Subdistrict	Policy implementers at subdistrict level ranging from CEOs managers, nurses and doctors implementing policies aimed at UHC as well as providing direct patient care	3	12	8	**23**
PHC facility	Policy implementers including operational managers and staff in PHC facilities implementing policies aimed at UHC as well as providing direct patient care	-	19	16	**35**
Total		**5**	**37**	**29**	**71**

## Results

Detailed findings of study were published in papers [10, 25, 26, 29, 30], and some additional results will be presented here. In this study we analysed how the CIT central tenets, Information, Motivation, Power, and Resources and interactions of these affected UHC policy implementation. We also analysed the data inductively.

One of CIT`s key assumption is that factors influencing implementation are interactive. The influence of any factor whether positive or negative depends on the particular context. The theory distinguishes a set of core constructs or concepts related to the actors involved which jointly contribute to implementation. Core constructs are;

## Motivation

The level of importance the actors place on a policy and the degree to which policy contributes to their goals and objectives affects implementation. If actors have low motivation, they may ignore implementing the policy. Examining motivation helps to understand the perspectives of implementers, their belief system, value priorities and perception of the importance and magnitude of specific problems often revealing root causes of implementation barriers [16]. Participants cited having a high intrinsic motivation like being nurses because it’s their calling. Extrinsically, they explained how the shortages of staff, equipment and infrastructure prevented them from implementing policies successfully. Policy makers at provincial level perceived the situation differently. They were confident that all their facilities had sufficient equipment. Participants revealed the following;

But I’ve been here for a long time. Uh it`s just the environment that we’re working in with all these shortages. You can see, it’s frustrating-PHC Manager round 1

Yes, the staff morale is gone, and you see the people are leaving so quickly, some people come here, get appointed, and after two months they decide to leave. You see all the doctors that were appointed in January, there is about nine of them, already gone- sub district Manager round 2

## Information

Successful policy implementation requires that those involved have sufficient information including technical knowledge of the matter at hand, levels and patterns of communication between actors. For example, do those responsible for implementation actually know with whom they should be working and who the policy should benefit? Do they know which department is assigned to lead the implementation and how the programme will be monitored? How is information and communication between actors coordinated? Have guidelines been developed and are they readily available? [11, 16]. For a policy to be successfully implemented, ideally implementers should be involved in policy development and if not possible, the policy should be communicated well and understood. Participants revealed the following to highlight policy communication gaps

I have never heard of obstetric ambulance. It would be great to have them but I have never heard about them or seen them here-PHC Nurse round 2

I had not heard of this before, and there wasn’t anything in writing from National, so it was a big jump in faith. But we’ve gotten used to it and we are doing it. We were conservative about it. They said employ fifty and we then ended up with thirty-two, which I think is okay- it’s been quite difficult. We have had to think up things. -District Actor round 2

## Power

Who is empowered to implement policy and to what degree? Power may derive from formal sources such as a legal system e.g., appointment or from informal sources such as charisma or being an elderly. The power to appoint or dismiss staff, to mobilize resources or adapt policy is essential for successful policy implementation. The dissonance between responsibility and authority proved to be one of the implementations stumbling blocks. Participants revealed the following;

The budget is not with the district, the budget is with the head office. I have no power over that, but there are things we need that impact negatively on the quality of care. I cannot do anything- District Actor round 1

Some of the problems are supposed to be resolved by the district. Our hands are tied. I could solve some of the problems but I have no power, so I have to refer to the district-PHC Manager round 2

## Resources

Having adequate resources for the intended action is important for actors to realize policy implementation goals. Resources provide the capacity to act [31]. The relevance and availability of resources influence the actors motivation which in turn influences the whole policy implementation process [19]. Participants revealed the following;

And then supplies, we are running out of supplies. So how are we going to meet NHI standards, we haven’t got the supplies? Like in our clinic for instance, we haven’t even got anti-bacterial soap-sub-district Manager round 1

We don’t even have a defibrillator but that is a requirement of the current national core standards. We do not have the basic equipment-PHC Manager round 2

Because the resources you need are not available to implement those things. We are talking about NHI for the past two, three years now- you see. But very little implementation, just talking-particularly with the additional resource needed. So, NHI is good for the standard of care but to implement those you need the resources. It says like for the medical ward, you need these number of square metres between beds or this number of beds in a ward. And currently we are far behind from complying with that standard. -sub district Manager round 1

## Interactions

Interactions predict the level of collaboration among and between actors which in turn influence policy implementation. They must be considered to further analyze barriers to implementation. These interactions can take different forms from cooperation, passive cooperation, forced cooperation, opposition or joint learning. In turn, actors collaboration depends on how they perceive the problem being addressed as a priority, how convinced they are that there is an acceptable solution, that taking action now is in own best interest and if they have implementing capacity [11, 16]. Specifying the above constructs facilitates the development of tools to measure the level at which each of the core construct contributes or hinders implementation [16]. Passive cooperation was prevalent during the first round and this changed into mild opposition, with some actors refusing to keep on covering up during audits. Audit driven compliance with national core standards was revealed as a big issue by most of the participants. Participants revealed the following;

I mean they tell us there is an audit. Clean the clinic. We have to clean the clinic despite sending them several reports that we do not have a cleaner. I told them it is not in my job description to wash the walls and make the clinic look nice so National and Province can come here and say the clinic looks beautiful. They must see it for what it is. I will not do that. It is not in my job to clean-PHC Nurse round 2

You understand, because next week we’re having an audit. That is why they are now sending us policies in bits and pieces. No, I will just leave it as it is. Why must I cover up? Every time we get audits, our facility covers up for a lot of things to say, we are fine, we are okay. I can tell you, we’re not okay, things are not working-sub district Manager round 2

**Fig. 1:**
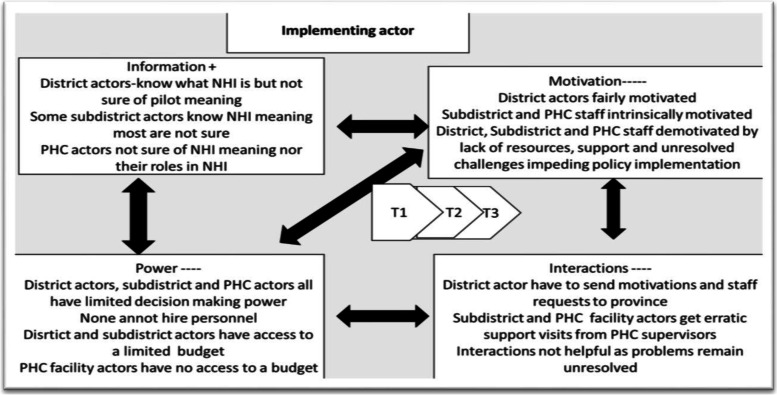
Policy Maker CIT tenets. T1 = time 1 of data collection; contextual mapping T2 = first round T3 = second round

**Fig. 2:**
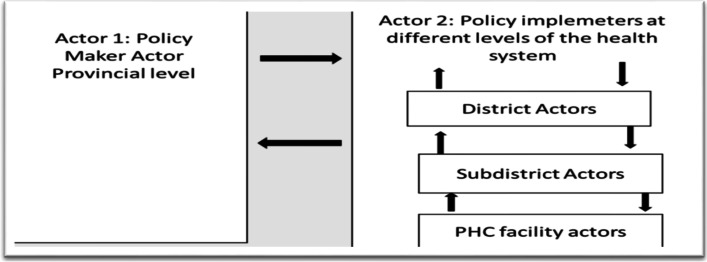
Interaction process

**Fig. 3:**
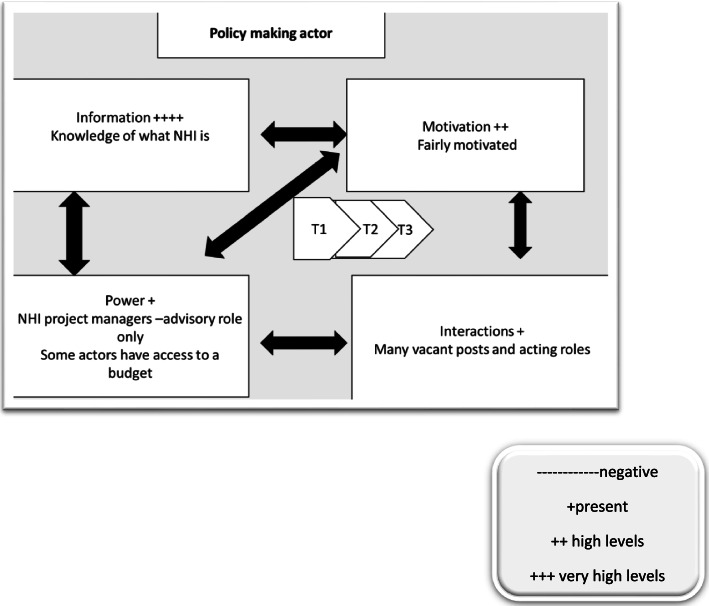
Policy Maker CIT tenet

A summary of findings from the overall study with regards to CIT tenets are presented. See Table 4 below; (Figs. 1, 2, 3)

**Table 4:** Summary of Findings

Core CIT construct	Policy maker	Implementer
**Information**	Fully informed and aware of NHI policies and intended benefits	District and senior staff aware but many frontline actors have little understanding of their roles [10]
**Resources**	Some actors had access to budgets	District, subdistrict and facility staff cited lack of human, material and infrastructural resources to fully implement policies [10, 26]
**Motivation**	Some actors were new appointees to drive the NHI policy implementation and were generally motivated	District and subdistrict actors were demotivated by dysfunctional systems particularly supply chain [10, 26]. Facility staff were demotivated due to lack of resources, dysfunctional systems including employee performance and management systems and lack of support from above [10, 26] Facility staff were demotivated due to being caught in between with pressure from both patients and supervisors [10, 26] Facility actors were demotivated due to longstanding problems that do not get resolved [10, 26]
**Power**	Some actors had access to budgets and power to appoint personnel Other functions are only advisory in nature e.g., NHI project Manage	District, subdistrict and facility staff all cited no power to appoint staff [10, 26] Facility actors have no access to budgets [10, 44] District and subdistrict Managers cited having limited power and financial delegations [10, 44] According to Elmore funding affects implementation [45]
**Interactions**	Actors were housed in one building and had regular meetings though many posts vacant	PHC supervision not frequent enough [10, 26] PHC supervisor not able to solve facility challenges [10, 26]. PHC supervision seen as policing and not supportive [10, 29] National core standards failing facility staff for issues beyond their control [10, 26] According to Elmore, authority relationships affect implementation [45]

## Discussion

This paper applied a two actor CIT theory to analyse 71 interviews from a South African NHI pilot site. This paper contributes to implementation studies through the testing of the contextual interaction theory in a UHC setting, not done before, to the best of our knowledge. The study aimed to depict how actor characteristics (Information, Power, Motivation, Resources and Interactions) influenced UHC policy implementation process in one pilot district in South Africa [10]. We utilized CIT factors to understand how the policy-practice gap came about. We also expand the theory by adding one more core variable: leadership to information, motivation, resources and power and interactions [29]. Leadership Interaction is defined as an occasion when two or more people communicate with or react to each other [32] particularly between senior and junior actors, including supervisor-supervisee relationships.

CIT captured the implementation challenges in this context very well and our findings could fit into the central tenets: information, motivation, power, resources and interactions. Our findings also replicated interactions of information, motivation, power and resources as well as human factors, perception, response and motivation [10, 26]. Many of the challenges cited, though they fitted into these central tenets, pointed at a leadership gap. Lack of knowledge of policy (information), lack of resources like equipment, lack of power to mobilize the needed resources and lack of support from the top, were repeatedly cited as a challenges by implementing actors [10, 26, 30].The unresolved challenges themselves demonstrate a leadership gap that is compounded by the non-responsiveness of those who had the power, another leadership gap.

### Adding and highlighting a fourth construct leadership (meaning supervisor supervisee interactions)

The tenet Leadership, is not explicitly represented in CIT [33]. This study also serves to illustrate CITs flexibility to contexts by adding a fourth variable, namely leadership interactions, to the CIT core variables, motivation, information, resources, power and interactions of these.

**Fig. 4:**
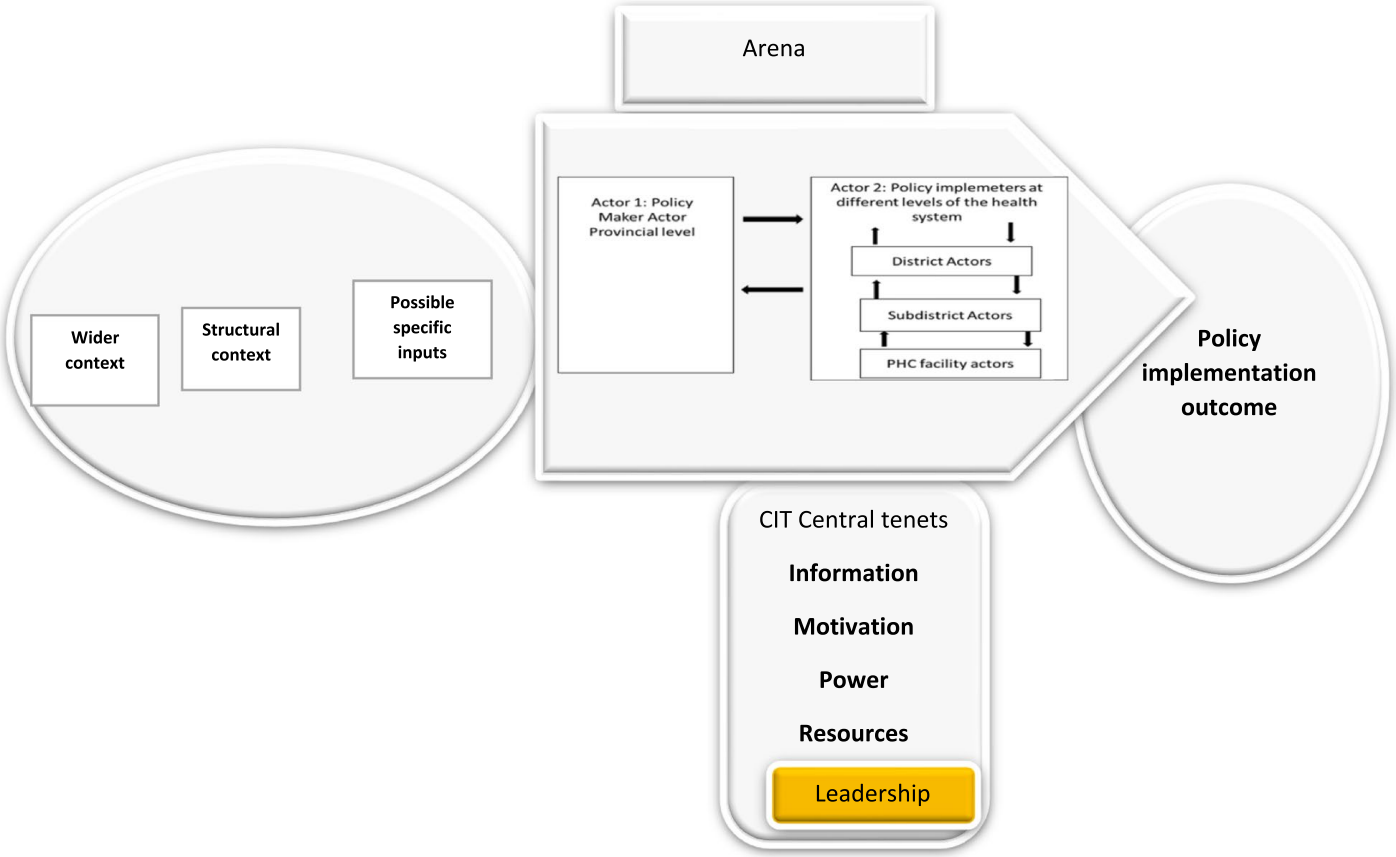
The modified CIT process model which includes the fourth construct Leadership

In all the analysed cases for this study and published papers [10, 25, 26, 29, 30] as well as the findings presented above, it became apparent that ignoring leadership in policy implementation could leave out important contributions, that illustrate how leadership influences the implementation process. One of the UHC policies that was under implementation was an ideal clinic. An ideal clinic is defined as a clinic with good infrastructure, adequate staff, adequate medicine and supplies, good administrative processes and adequate bulk supplies that use applicable clinical policies, protocols, guideline as well as partner and stakeholder support, to ensure the provision of quality health care services to the community [34]. Leadership in implementation is key and critical in as far as it affects resource availability, motivation and information. Most of the actors' motivation was affected by longstanding unresolved challenges confirming the leadership gap. Our research revealed chronic staff shortages, material, human and infrastructure, leaking roofs, unrepaired toilets, supply chain woes, supervision challenges, non-responsive leadership and long waiting lines [10, 26, 29], demonstrating an implementation failure. There seems to be a lack of people with visions of what ought to be (ideal clinic as an example), people who can inspire others to strive forward, motivating them, aligning and mobilizing resources to ensure successful implementation [35]. We also would like to acknowledge that there are many people in the health system. with leadership qualities that expressed being constrained by the work, organizational environments and cultures. The role of organizational culture in this regard needs to be explored. Leaders inspire, leaders motivate, leaders communicate and leaders align and mobilize resources to ensure effective policy implementation. To accurately reflect the SA UHC context, we propose adding leadership as a core CIT tenet to become: Information, Power, Motivation, Resources, Leadership and Interactions of all these see Fig. 4 below.

### The critical role of leadership in implementation

The critical roles of leadership-the human factors and our proposal to adapt the CIT tenets to (information, motivation, power, resources, leadership and interaction of these) are discussed.

### Leadership definition

Leadership Interactions are defined as an occasion when two or more people communicate with or react to each other [32], of particular importance in our study is the formal relationship between managers and subordinate actors including supervisor-supervisee relationships. In the context of our study, meaningful leadership interactions are formal relationships in the health system, implementing actors can fall back on for support, role clarification, motivation and problem solving. Effective leaders have decision making power and financial delegations to unlock resources and solve existing problems and challenges impeding implementation [29, 36]. There is a two-way open communication where leadership is present. Leaders have a vision, inspire subordinates and align and mobilize resources to ensure successful policy implementation [36]. The chronic resources challenges and lack of support cited by implementing actors’ points at leadership gaps [10, 26, 37]. Negative leadership interactions were revealed as non-responsive and blame and punishment of subordinates whenever things went wrong. That in turn created a chain of responses by implementing actors, the human factors in the system, creating an added layer of implementation barriers [10].

Policy implementation failure has been repeatedly associated with a lack of leadership [4, 15]. According to Meyers and Dillon 1999, something happens within the organization that can either halt the process of implementation or speed it on its way. That something seems to be associated with organizational leadership [10, 15, 29].

### Contribution to the literature

- Empirical research discloses implementation as a significant impediment to change. Understanding what facilitates or hinders implementation is critical as many countries strive towards universal health coverage: these findings advance our understanding of how to effectively implement UHC policies in a context similar to South Africa
- Policy implementation is a complex process. A theoretical framework is a lens through which policy implementation can be understood. The literature is awash with many implementation theories. Understanding implementation theories that capture implementation experiences of actors in a particular context aids in reducing policy-practice gaps. Our study has demonstrated the utility of CIT in UHC policy implementation context-South Africa
- Contextual Interaction Theory was developed in a water governance and policy implementation context. To the best of our knowledge, this is one of the first studies that applied CIT in a UHC policy implementation context, assessing its utility and identifying any short-comings. We identified leadership as a critical factor, actors repeatedly alluded to as affecting implementation. This factor however is not explicitly identified as a central tenet in CIT, hence our proposal to include this and improve the utility of CIT in contexts like South Africa. Adding leadership to these central tenets is our proposal to make CIT useful in contexts similar to South Africa. We propose the central tenets to become motivation, information, power, resources, leadership and interactions of all these.
- Contexts matter a lot in implementation. Theoretical frameworks are important road maps in policy implementation and analysis. CIT is a simple but very useful theory and framework that condenses reality into less detailed but informative elements (information, motivation, power, resources, interactions of these). Our contribution to literature is an adapted CIT theory that fully captures policy implementation experiences of actors in a UHC context.

### Limitations and strengths

One of the limitations is that our study was carried out in only one pilot district though our findings concurred with studies from other pilot sites. In future, studies ought to be carried across multiple pilot districts. Funding however, is a challenge that makes such multiple-sites studies not feasible. Another drawback is that UHC policies were the focus of our study and so we did not touch on other national policies that were also in implementation at the same time. One initiative that was rolled out in the country including pilot districts was campaign on Accelerated Reduction of Maternal Mortality in Africa (CARMMA). The impact of CARMMA and similar policies that were rolled out parallel to UHC policies, on the outcomes observed, cannot be disentangled. This short coming is difficult to overcome in observational studies.

UNITAS on the other hand, was one of the first systems set up to document, track and monitor UHC policy implementation in a low- and middle-income countries. That way, utilizing a theory of change approach, engaging both policy makers and policy implementers, the experiences of these actors could be captured over time-5 years. Empirical examples of challenges that hindered implementation and factors that facilitated success could thus be captured, providing a wholistic picture of what goes on in a South African UHC policy implementation context. On the whole, the qualitative methodology in conjunction with CIT analytic framework, allowed us to answer the why and how questions of policy implementation, thereby getting deeper insights. Finally, to the best of our knowledge, the utility of CIT has not been tested in a UHC coverage context before-making our study one of the first ones to assess CIT utility in a low-to middle-income UHC context.

## Conclusion

### Case for leadership as an additional CIT tenet

CIT proved to be a useful framework for analysing data from a UHC implementation site. The CIT central tenets Information, Motivation, Power, Resources and Interactions were observed, alluded to by all actors and their utility in policy implementation were replicated in our study. One aspect that dominated the South African policy implementation context was leadership. This leadership gap has been revealed in literature before as an impediment to successful policy implementation [35, 36, 38–40]. Our study findings repeatedly pointed at this gap. Leadership was associated with information, motivation, resources, power and interactions of these. Leaders are not satisfied with the status quo [35].Without a functional basic health care system, all health and its related targets are in jeopardy [41]. For this to happen, we reckon leadership is needed. Current evidence points to gaps between policy and practice without necessarily explaining how this comes about [42, 43]. The importance of leadership in policy implementation has been revealed by our study, hence it cannot be overemphasized. The utility of CIT as a framework in these contexts, can therefore be enhanced by identifying the importance of leadership in implementation and incorporating leadership as a central CIT tenet.

## Data Availability

Data and material from this study cannot be provided publicly due to ethical obligations to protect anonymity of participants. As stipulated in the participants “informed consent form,” data access is limited to members of UNITAS research team. For further information related to data, please contact the corresponding author.

## Abbreviations

CIT: Contextual Interaction Theory
UNITAS: Universal Coverage in Tanzania and South Africa
UHC: Universal health coverage
SA: South Africa
